# Smokers’ Views on Personal Carbon Monoxide Monitors, Associated Apps, and Their Use: An Interview and Think-Aloud Study

**DOI:** 10.3390/ijerph15020288

**Published:** 2018-02-07

**Authors:** Aleksandra Herbeć, Olga Perski, Lion Shahab, Robert West

**Affiliations:** 1 Research Department of Behavioural Science and Health, University College London, London WC1E 7HB, UK; lion.shahab@ucl.ac.uk (L.S.); robert.west@ucl.ac.uk (R.W.); 2 Research Department of Clinical, Educational & Health Psychology, University College London, London WC1E 7HB, UK; olga.perski.14@ucl.ac.uk

**Keywords:** smoking cessation, mHealth and eHealth, intervention development, carbon monoxide, smartphone, qualitative study

## Abstract

Smartphone-based personal carbon monoxide (CO) monitors and associated apps, or “CO Smartphone Systems” (CSSs) for short, could enable smokers to independently monitor their smoking and quitting. This study explored views and preferences regarding CSSs and their use among 16 adult, UK-based smokers. First, semi-structured interviews explored participants’ expectations of CSSs. Secondly, a think-aloud study identified participants’ reactions to a personal CO monitor and to existing or prototype apps. Framework Analysis identified five themes: (1) General views, needs, and motivation to use CSSs; (2) Views on the personal CO monitor; (3) Practicalities of CSS use; (4) Desired features in associated apps; and (5) Factors affecting preferences for CSSs and their use. Participants had high expectations of CSSs and their potential to increase motivation. Priority app features included: easy CO testing journeys, relevant and motivating feedback, and recording of contextual data. Appearance and usability of the personal CO monitor, and accuracy and relevance of CO testing were considered important for engagement. Participants differed in their motivation to use and preferences for CSSs features and use, which might have non-trivial impact on evaluation efforts. Personal CO monitors and associated apps may be attractive tools for smokers, but making CSSs easy to use and evaluating these among different groups of smokers may be challenging.

## 1. Introduction

Only a small minority of smokers use evidence-based support during quit attempts, such as face-to-face behavioral support or pharmacotherapy [1,2]. This calls for the development of new, acceptable cessation aids. Smartphone apps have been shown to be acceptable to some smokers, although evidence for their effectiveness is lacking [3,4,5,6]. A potential new cessation aid could involve smartphone-enabled personal testing of carbon monoxide (CO) levels as part of quitting or cutting down. In accordance with principles of user-centered development of complex digital programs [7,8], this study aimed to understand the needs and preferences of potential users to inform further development of and research involving such interventions.

CO is an invisible, odorless, but toxic gas that is formed during tobacco smoking and can be measured in the exhaled air of smokers using CO monitors [9,10]. CO levels, measured as particles per million (ppm), can help distinguish between different levels of smoking, with levels below 10 ppm commonly used as indicators of abstinence [11,12]. However, light smoking (e.g., as part of harm reduction) may result in readings of 5–9 ppm [13]. There are several important benefits of CO testing. First, it acts as a non-invasive measure of harm from exposure to active and passive smoking [14,15,16], which conveys more information than that provided by the number of cigarettes smoked alone (e.g., CO levels can be affected by the intensity with which cigarettes are smoked). Exposure to CO, also in the form of pollution, is associated with incidents of and mortality from stroke and other cardiovascular diseases [17,18]. Second, CO levels are not affected by concurrent use of nicotine-containing products such as e-cigarettes or nicotine replacement [9]. Due to the body’s ability to rapidly eliminate CO, the CO half-life is about 4.5 h [19], with CO levels returning to normal within 24 h since last cigarette. Hence, the temporal applicability of CO testing is limited, but it can be used to trace progress with cutting down and harm reduction [20]. Additionally, CO results may be affected by factors such as smokers’ health status (e.g., asthma or COPD) [21,22] or exhalation speed into the CO device [23].

The assessment of CO levels has commonly been used as part of a range of smoking cessation programs. For example, CO monitors have been used as diagnostic or educational tool in clinics or during health promotion campaigns [24], as biochemical confirmation of abstinence from smoking during treatment [9,12] or in research studies [11,25]. CO testing has also been identified as a valuable monitoring and feedback component of effective stop smoking programs [15,26,27]. CO testing can further benefit cessation through making the health impact of smoking more salient [15]. However, as the first-generation CO monitoring devices are costly and relatively large, these have until recently been available only in clinical or research settings, with limited accessibility for individual smokers.

**Figure 1:**
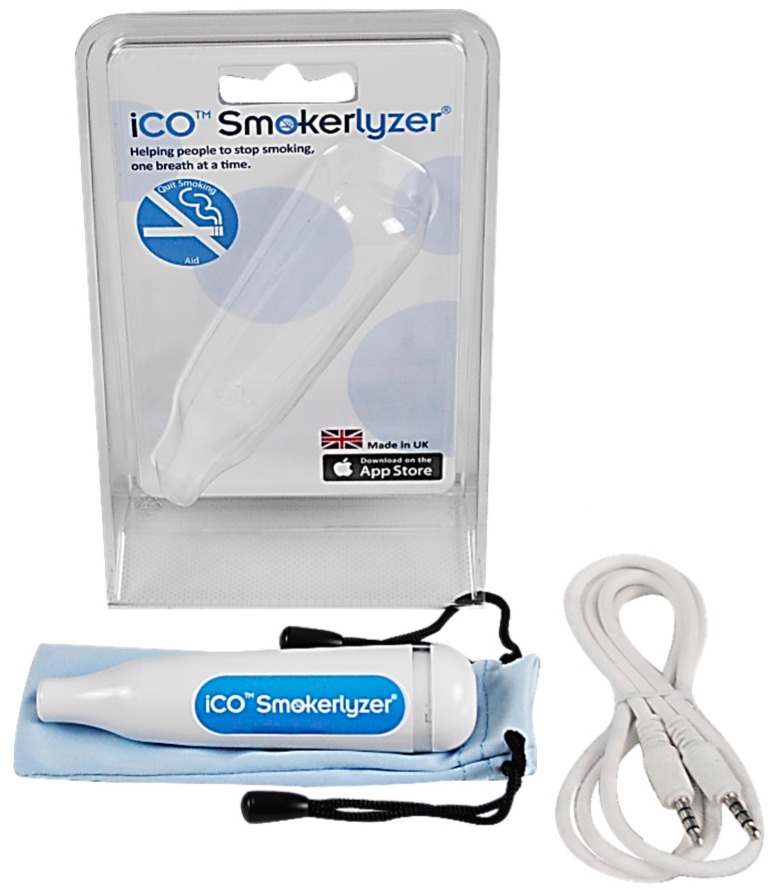
iCO™ Smokerlyzer^®^ developed by Bedfont^®^ Scientific Ltd., which was used as a prompt in the study to elicit views and preferences regarding a personal CO monitor and its potential use. The device uses a cable to connect to a smartphone through the phone’s audio jack. Images sourced from [40] with permission from Bedfont^®^ (© 2017 Bedfont^®^ Scientific Ltd.).

In the future, individual smokers could access CO testing outside of clinical contexts thanks to the emergence of smaller and more affordable CO monitors [28]. The iCO^TM^ Smokerlyzer^®^ manufactured by Bedfont^®^ Scientific Ltd. (Harrietsham, UK) is currently the only such device available for purchase. It connects to smartphones and requires a dedicated app to compute and display CO levels (Figure 1). Personal CO monitors could allow smokers to independently monitor and track CO levels and thus progress towards quitting, cutting down or harm reduction (e.g., maintaining a certain CO level, e.g., <10 ppm). This might be especially relevant for smokers who are not willing or able to interact with healthcare professionals as part of quitting (indeed, only around five per cent of smokers access stop smoking services) [2]. They could also help smokers achieve other pre-defined goals, such as reaching particular CO levels, and provide momentary feedback on behavioral outcomes, which are important self-regulatory and behavior change techniques (BCTs) [29] in both smoking cessation [30,31] and other domains [32,33,34]. Furthermore, preliminary research suggests that personal use of CO monitors is acceptable, valued and potentially highly motivating for smokers [13,15,35,36].

There are several important clinical, research, and practical advantages to using CO monitors that connect to smartphones and associated apps (later referred to as “CO Smartphone Systems”, or CSSs). Smartphone adoption is at 85% in the UK and growing [37]. Research suggests that over half of US smartphone users have downloaded at least one health app, although actual usage may still be low [38]. With regards to smoking apps, very few English smokers reported using a digital tool in their most recent quit attempt [39]. Nevertheless, smartphones offer a range of possibilities in creating dedicated apps that could harness incoming data from CO monitors to record, display, or otherwise manipulate information as part of complex behavior change interventions. Such app interventions could not only provide CO results accompanied by verbal or visual feedback, but also additional theory- and evidence-based features and advice aimed at increasing motivation, self-efficacy, or risk appraisal [15,35]. Furthermore, with the emergence of new technologies and programming solutions, there is also scope for personalization and interaction; integration with other platforms, data sources (e.g., other health indicators) or social media (e.g., buddy systems); and implementation of other interventions (e.g., contingency management programs [35]). Smartphone apps are also valuable research platforms, enabling efficient data collection and sharing, as well as testing of new design concepts through observational studies, A-B testing, factorial studies, and randomized controlled trials [7].

Although a number of studies have been conducted to elicit smokers’ views and preferences for digital cessation programs, including smartphone apps [41,42,43,44], limited research exists on CSSs. Little is known about the views of potential users on personal CO monitors, associated apps, and their use, all of which could impact on satisfaction, uptake of and engagement with CSSs, and eventually also on effectiveness [41,45]. This study aimed to address these gaps. The study is nested within a project that follows guidelines on the development of digital interventions [7], the Medical Research Council’s Guidance on the development and evaluation of complex interventions [46] and the Person-Based Approach to intervention development [8,47]. These approaches advocate the process to be iterative and cyclical, involving needs assessment with initial design concepts and research methods consulted among smaller samples of potential end-users, and iteratively adopted before being used in larger or more definitive studies.

## 2. Materials and Methods

### 2.1. Design

The study involved semi-structured individual face-to-face interviews and a think-aloud procedure [44] involving a personal CO monitor and existing and prototype apps. Due to the progression of the larger project, the interviews were conducted in two phases (eleven in 2016, and five in 2017). The first eleven interviews aimed to inform a new app prototype. The last five interviews in 2017 were conducted to ensure that saturation was reached, and were part of a study that involved the interview followed by piloting of methodology of home-based use of a personal CO monitor and the new prototype app (findings from the pilot are not reported as data collection is ongoing). Data from 2016 and 2017 interviews were analyzed together and are reported here. The study was approved by Research Ethics Committees at UCL (Project IDs: CEHP/2013/508; 6212/008).

### 2.2. Participant Recruitment

Participants were recruited through online advertisement and internal UCL channels (e.g., mailing lists and posters). Participants were invited to an interview study at UCL that aimed to understand smokers’ preferences and views on apps that connect to personal CO monitors as tools to support smoking cessation or reduction. The inclusion criteria were: (1) being 18 years or older, (2) a current daily smoker interested to quit, (3) owning a smartphone and be interested in using a stop smoking app, (4) be fluent in English, and have good or corrected vision. Additionally, participants recruited in 2017 also had to (5) be smoking 10 cigarettes/day, (6) have an Android phone and (7) be interested in testing the new CO monitor and app at home for a week while trying to cut down on smoking. Recruitment and data collection for the interview study stopped after data saturation was reached, meaning that no new themes or issues were arising from the data collected through additional interviews [48,49]. As the interviews were in-depth and focused on specific phenomena, saturation was reached after 16 participants.

### 2.3. Interview Procedure and Materials

All participants provided informed consent before the study initiated, and their data were labeled with individual participant codes to protect their identity. Participants completed a survey that assessed their history of smoking, quitting, and use of cessation aids (those ever-using stop smoking services or cessation medications, e.g., nicotine replacement therapy or on prescription medications, were classified as “ever users of evidence-based support”), prior use of CO monitors, as well as prior use of and current interest in smartphone cessation support. Interviews lasted between 50 and 90 min. Fourteen interviews were conducted by the first author, and two of the 2017 interviews were conducted by trained research assistants following detailed instructions. Participants from the 2016 interviews were reimbursed with £30 Amazon gift vouchers, and those in 2017 with a £100 Amazon gift voucher for participation in the week-long study.

All interviews followed a semi-structured interview guide and used a range of prompts, including visual materials (i.e., apps, app prototypes or designs that are being developed, and which are not complete interventions), which facilitated discussion of a range of possible future features and designs. The interview guide was also created to elicit information that could be useful to future manufactures of CO monitors, developers of associated apps, as well as researchers who may evaluate CSSs. Core sections of the interviews were common across 2016 and 2017, but a few changes were introduced in the second phase reflecting project progression. Appendix A outlines the interview topics covered in 2016 and 2017 (Table A1) and the prompts used (Table A2).

The interviews were divided into the following sections, and the interview guide was structured to explore these issues in depth: (1) current smoking patterns, experiences and views on smoking, quitting, and cutting down; (2) prior experience with CO testing, and with any cessation or health apps; (3) preferences for and expected use of hypothetical personal CO monitors and associated apps; (4) a think-aloud involving the iCO^TM^ Smokerlyzer^®^ developed by Bedfont^®^ Scientific Ltd. (Figure 1), which was purchased for the study; and (5) a think-aloud procedure, during which participants freely explored and said out loud their thoughts and impressions about provided apps, app prototypes, or designs. The apps used in phase 1 included the Smokerlyzer^®^ app which accompanied the iCO^TM^ Smokerlyzer^®^, developed by Bedfont^®^ Scientific Ltd., available in the Apple and Google Play app stores; two prototype apps (V1–V2); and designs created by the team at UCL (phase 1). Phase 2 included a new UCL prototype app (V3), informed by findings from phase 1, in addition to the other apps. App designs were only included in phase 1, as they helped to inform the new app prototype. After initial analysis, it became clear that their use did not contribute additional theoretical or practical considerations beyond those already emerging from other parts of the interviews.

The iCO^TM^ Smokerlyzer^®^ does not include replaceable mouthpieces, and so each device could be used by only one participant. Due to limited supply of the iCO devices it was not feasible to conduct CO testing during the interviews at that stage of the project. Finally, in order to obtain insights that could generalize to future CSS and to a wider group of smokers who might access CSS without professional support in the future, as well as to gain more aspirational and “naïve” views and expectations that were not constrained by shortcomings of the current CSS solutions or CO testing procedures, brief information about CO testing and monitors was provided only towards later stages of the interview.

Participant responses guided interview progression within each of the interview sections, but the interviewer ensured that all core topics were discussed. Impromptu probes were used to prompt elaboration. The interviews were audio-recorded and transcribed intelligent verbatim by a professional company.

### 2.4. Data Analysis

The analysis followed the principles of Framework Analysis (FA) [50]. FA is commonly used in applied health research [13,43,51], and one of its main advantages is that it supports a transparent and systematic analysis of large volumes of qualitative data, and is particularly suitable in projects with a well-defined participant sample and pre-determined themes, while also enabling emergence of novel themes [50,51]. The analysis was both deductive, whereby data were classified using a coding framework informed by the interview guide, as well as inductive, allowing novel findings to emerge. The analysis involved four stages: (1) familiarization through re-reading of transcripts, (2) identification of recurrent themes and subthemes using pre-defined and new codes, (3) development and refinement of a thematic framework through systematic indexing of transcripts, and (4) development of descriptive accounts and creation of explanatory frameworks. All data were analyzed together, regardless of the context in which they emerged. Data were sometimes coded to multiple codes. As the current study was exploratory, all participants’ accounts were treated as true and important. A realist epistemological perspective was adopted [52]. Data analysis was conducted in NVivo 11.

The final coding framework was agreed through three rounds of iteration and internal validation. First, the first and second authors independently coded 11 and three interview transcripts form phase 1, respectively. The resulting coding frameworks (v1a and v1b) were compared. The first author then prepared a revised framework (v2), which was applied by the second author to two new interviews. Following discussion and adjustment, a final version of the thematic and coding frameworks (v3) was created by the first author and applied to all transcripts. Summary tables with the coding framework and exemplar interview quotes were checked for internal validity and consistency by the second author. Constant comparison [52] and deviant case analysis [53] were used to ensure internal validity. All participants were emailed a summary of findings and could provide additional comments, if they wished [54]. Four out of five participants from 2017 interviews replied that the findings reflected their experiences and views, and did not suggest any changes.

## 3. Results

### 3.1. Participants

The interviews were conducted with 16 adult participants (aged 20–51), of which eight (50%) were men, seven (44%) had prior experience of CO testing as part of cessation programs, and three (20%) had used stop smoking apps before (see Table 1).

### 3.2. Findings from the Interviews

Five main themes with several subthemes each were identified. The main themes are described below, together with illustrative quotes (the numbering of sub-themes is presented in brackets, with the corresponding labels reported in Table 2 and Table 3). Table 3 summarized design suggestions for personal CO monitoring devices and associated apps, as emerging from themes 4 and 5.

#### 3.2.1. Theme 1: CO Testing—General Views and Motivation to Use

Except for few participants, especially those with suboptimal prior experiences of CO testing, many participants were keen on using CSSs, and viewed them as a valuable and promising novel tool for self-exploration and smoking cessation, and one that offered convenience and independence from healthcare professionals (1.1.) (“*It’s exciting to be part of something new, and also if that helps, anything that helps kicking of a habit is welcome”—P7*). CSSs were expected to be especially helpful for increasing motivation to quit or to remain abstinent (1.2.) (“*…psychologically I’m going to feel better because I want to see the results showing me that I’m you know, getting healthier in a sense”—P14*), particularly by showing the health damage of smoking (“*I would continue to smoke normally just to see how big it’s going to get so I can frighten myself so I can stop.”—P9*). Some participants also believed that a CSS could become a long-term companion through the smoking and quitting journey (“*...in an ideal situation, I would have it just to use indefinitely, and I would start off and I would keep smoking for the first couple of weeks at least, start getting some feedback, building up a bit of a data pattern, and then I would sort of put it together with some other [stop smoking] approaches”—P16*).

Participants expressed additional reasons to use CSSs (1.3.). These included: interest in the “quantified self” (i.e., to assess and document in detail one’s behavior and its outcomes); the opportunity to learn new things about oneself (“*I’m really excited, because again it’s something that will tell me something about me or my body, that I’m not aware of”—P7*); willingness to contribute to scientific research; having a new tech gadget, also to show it off among friends (“*I think, it would seem to me to be, like, a bit of a novelty, so that I’d, I’d imagine, sort of, doing it and showing my friends, being like, hah, look, I’ve got lower CO than you, or whatever”—P4*).

Nevertheless, some concerns were also raised with regards to the use of CSSs (1.4.). Some participants were concerned with the precision of CO testing, and with factors that may affect the results, such as type of cigarette smoked, timing and method of CO testing, co-use of other substances, or smokers’ characteristics (“*Well there will be still a trace of scepticism […] this is why I’d still try the 2 weeks’ challenge with the cigarettes, so I’ll try to smoke with people, I’ll try to smoke on my own on different days and just, I want to see if there’s going to be a difference in the level.”—P9*). Another concern was related to potential negative or undesired outcomes (1.5.), such as worries about high CO results (“*And it’s quite scary though to know how much is in my body. God, it’s quite scary.”—P6*)*,* or annoyance and discouragement when not seeing sufficient decline in CO reading (“*…if you see that it’s not making a difference, you kind of feel a bit … demotivated.”—P8*). The interviews also revealed possible unintended outcomes, whereby low or moderate CO readings or goals for harm reduction (e.g., allowing smoking as long as the CO results stayed below 10 ppm) could be seen by some smokers as permissive and reassuring of continued smoking (“*You actually see what the middle is [on the CO scale] and if you’re in the middle I don’t think you would be very scared.”—P9*).

#### 3.2.2. Theme 2: Practicalities of CSS Use

Participants described different expected patterns of CSS use, for example depending on whether it would be part of a study or used as a commercial product (2.1.). Use during the study was associated with greater acceptance of registering personal details, sharing results with others, and using the app based on a pre-determined schedule (“*I’m assuming that this is just for the research purposes, I wouldn’t, if I was to be a general customer I wouldn’t be expected to have to put all these numbers in would I?”—P15*). In contrast, use of a CSS outside of the study was expected to be more *ad libitum*, for longer, or less frequently.

Participants also showed different preferences for CO testing depending on their smoking status (2.2.). While some participants were interested in documenting CO levels across different experiences and stages of quitting (“*I would like to use it in situations when I am smoking more, for example sometimes you go through a patch in your day when you are overly stressed”—P8*), others had clear preferences for testing when they expected the results to be either low (e.g., to confirm and reward abstinence), or high (e.g., to scare themselves into refraining from smoking) (“*I’ll probably only do it when I know that I haven’t smoked in a while.”—P4*).

When discussing location of CSS use (2.3.), about half of participants wanted to use the CO monitor only in private, and ideally at home (“*Err, yeah looks great, I suppose that, I guess you wouldn’t carry it round, I suppose it’d be something that you’d just have at home.”—P4*), while others seemed happy to use it in public or in front of friends, sometimes also strangers (“*I wouldn’t whip it out at a pub necessarily, but yeah, with friends why not?”—P7*). Participants also had different views on sharing the device (2.4.). Some participants thought of the CO device as a very personal item, never to be shared, while others were very keen to share it with friends or family as a way of encouraging them to quit, to compare results, or just to demonstrate the CSS’s capabilities (“*I could carry it around with me and blow into it, and I could probably pop it out and show somebody I would see smoking and say, ‘Test your breath as well and see how you’re doing’”—P3*).

Participants also had different preferences for the timing and duration of CSS use (3.3.). Many considered morning and evening testing as the most probable times, especially for home-only testing. However, some participants were keen to use CSSs throughout the day and in a range of situations (“*I mean I imagine you’d do it maybe 3 times in a day perhaps or twice in a day, the start and the end of the day or something.“—P15*). While some participants perceived CSSs as a potential aid during a specific quit attempt, others expected to use CSSs over several months or even a year, for example to document and learn from their smoking and quitting journey (“*I want something that I can charge and use and at least for, because I mean you can’t really say you’ve quit smoking unless you have like at least six months behind you, six months to a year.”—P5*).

Some participants mentioned several potential barriers to CSS use (3.6.), especially after demonstrating the iCO^TM^ Smokerlyzer^®^ and discussing its usability. These included: annoyance or anticipated inconvenience related to the action of blowing into the CO monitor or needing to connect it to a phone for each test; dislike for carrying and using the connecting cable; dissatisfaction with the irreplaceable battery; or embarrassment of using the current device in public (“*I would use it more if it didn’t have, probably, the wire. […] I don’t think a lot of people would want to take their phone out and plug that in in front of someone to show […], I’d want to do that, definitely at home, in private, yeah.”—P3*). Finally, some participants saw little relevance of using CSSs when smoking lightly or successfully abstaining, or anticipated losing interest in CO testing long-term (“*I guess […] it will only work for people who are smoking quite heavily, because if you’re, if you’re smoking quite light then […] you’re not going to see huge improvements.”—P8*).

#### 3.2.3. Theme 3: Factors Potentially Affecting Preferences, Views and Engagement with CSSs

Several factors emerged as relevant to understanding participants’ preferences, views and expected engagement with CSSs. One of these was participants’ smoking profile (3.1.), and especially patterns of smoking (i.e., regular smoking vs. smoking primarily during workdays, in the evenings, or when stressed), dependence levels, and the role that smoking played (e.g., as a habit, mood regulation, or socialization). Participants considered these when reflecting on their preferred timing or context for CO testing, such as expecting to use the CSS when normally smoking a cigarette. Another was the perceived barriers to quitting (3.2.), particularly: low capability and self-efficacy, low motivation, and other concerns, such as weight gain. Participants expressed preferences for apps that would address these barriers, but particularly had high expectations for the CSS to help them raise and maintain motivation to initiate quitting and prevent relapse (“*I always I give up for like two three months and then take it back up again, so maybe you know bits of, I don’t know, something about the long term I suppose would be quite useful.”—P4*).

Participants’ views on, and plans for quitting (3.3.) seemed also relevant. Some focused on a specific quit attempt, while others viewed quitting as a long-term journey involving some self-discovery and experimentation. Participants also differed in their preferences and plans for quitting, and particularly with regards to the approach (cutting down or quitting abruptly), the levels of support (e.g., no support, minimum advice, or intensive support from healthcare professionals), and timeframe (near or distant future). These preferences and plans were reflected in participants’ preferences for CSS features and expected use (“*I think for me at least it will take at least a month before I cut back to say less than ten a day, at least a month, and then another three months before I can go for five a day. So having that constant log of information would be really great.”—P1*).

Prior experiences also emerged as important. First, almost all participants had some experience of using digital devices (e.g., wearables) or health apps, but few had ever used stop smoking apps (3.4.). Some participants voiced preferences for using CSSs that included features that they enjoyed elsewhere (e.g., use of targets or automatic assessment of pulse to tailor advice on running, badges and missions in other quit smoking apps), and often compared the designs and functionality in the demonstrated apps with other, familiar apps. Participants also described their habits or preferred interactions with other programs, which they were also generalizing to potential CSSs, such as skipping tutorials, reluctance to register with personal details or to share data, or limited patience for apps that ‘freeze’ (“*I think maybe a little timer somewhere or a sand timer or something, ‘cos if I was at home I might have thought it had crashed, and I do that a lot, if an app doesn’t respond quickly I’ll close it.”—P10*). Second, many participants with prior experience of CO testing (3.5.) viewed CSSs as beneficial and important. However, one participant remembered being able to easily ‘cheat’ or manipulate the CO result, which made him more skeptical about the value of CO testing (“*I don’t think it’s that accurate. Because if you smoke just before you take the test you’re going to blow a high reading, but if you don’t smoke for, I don’t know, two or three hours beforehand and blow…”—P10*).

#### 3.2.4. Theme 4: Personal CO Monitor: Features and Qualities

Many participants were positive about the design (4.1.) of iCO^TM^ Smokerlyzer^®^, and praised its small size, good appearance and packaging, and low weight (“*It’s cute and like I think, yeah, it’s designed really well, it’s like something that goes, that you can put in your bag and doesn’t take up much space ...”—P2*). It was also favorably compared with first generation CO monitors among participants with prior experience of CO testing. The clinical appearance of the device was either seen as an advantage or disadvantage.

With regards to functionality and usability (4.2.), some participants were disappointed by the short battery life (warranty for 3 years, or for 200 readings), especially those who envisaged using a CSS long-term. Also, many participants felt that the cable connecting the device with the app was too long and that it negatively affected the appearance and usability of the device (“*Yeah, if it was the wire, I’d take it in my bag, I’d carry it around, but because I know that I have to use it with the wire, in my phone, it wouldn’t come out of the house, it would probably go in the drawer.”—P3*). The simple functionality was favored by many participants, but some were disappointed about results not being displayed directly on the device, and the reliance on connecting the monitor to the smartphone during each use. Some voiced preferences for the device to collect additional data, ideally automatically (“*I don’t know, maybe even put a hair sample in it or something, or saliva.”—P10*).

#### 3.2.5. Theme 5: CSSs Apps: Features and Qualities

Participants discussed a range of features and content of apps that could work alongside personal CO monitors, which are summarized in Table 3. CO testing journey was a central feature discussed (5.1.) Participants expected the CO test to be immediately accessible on app launch, to follow a quick and intuitive process with limited text on screen, and perhaps use of visuals or icons. They also preferred detailed, numeric results on screen (in particles per million), rather than a range. Many participants expected the result to be accompanied by brief feedback, including use of visual or color-coded scales, and by motivating information and tips on quitting and cutting down (“*I need to know what this [result] means, like what is normal and is this horrible, is this not so horrible? […] Yeah, I would like to have a record of this as well […] and if it gives information on how to cut down as well, yeah.”—P11*). Additionally, some participants were interested in collecting pertinent, contextual data on their individual CO readings to better understand their behavior and outcomes, such as quantity and timing of cigarettes smoked, use of cessation aids, context of CO testing (e.g., location, timing), or self-reported levels of stress, cravings or other emotions (“*My smoking is quite mood related […] I would like to do is that I can quickly record why I am having a cigarette and two, three things, how much was the craving […], what triggered this particular smoke and is there I can do something alternative to not go for this cigarette.”—P13*).

Other key desired featured included having access to a detailed and interactive infographics (5.2.) of CO readings, with many participants voicing preferences for interactive timelines, with adjustable timeframes, and the possibility to combine different pieces of information, e.g., about contextual data on CO readings (“*Yeah, and maybe sort of just a little bit more interesting or more integrated so it’s got sort of like, potentially like goals […] so it’s the sort of thing that suggests what you could do in the future…”—P4*). Participants were also interested in relevant factual content (5.3.). The concepts of CO and CO testing were novel and attention-grabbing for many participants, who were curious about learning more details about the scientific, health and practical aspects of CO measurements (“*I’m spending a lot of time on the information [in the Smokelyzer^®^ app], like I want to read through it, [...] Just by reading it I feel like I would smoke less cigarettes tomorrow just by reading it […]. amazing, I love the health section.”—P9*). Nevertheless, some participants were still expecting to see tips and advice on smoking cessation and reduction. A range of additional features (5.4.) was discussed, such as customizable reminders about CO testing, a possibility to set target CO levels or other goals, in-app rewards (e.g., badges), external rewards (e.g., certificates), as well as craving management aids (“*something about this app would help sort of fidget oneself away from wanting a cigarette”—P1*). There were also mixed views on sharing CO results with others, including on social media, with some participants keen to be part of an interactive peer support group, and others considering CO readings too personal to share.

While CSSs were praised as standalone programs, some participants believed that it would be beneficial to integrate them with existing face-to-face cessation programs (5.5.), include functionality allowing users to share their CO results with clinicians who may oversee cessation efforts, or at least provide contact to cessation experts (“*I think it needs to be part of a package rather than just its own thing, […] like you have to like forward along the readings and then like you go back like sometime later for like a follow-up”—P2*).

There were some mixed views on general app qualities and information architecture (5.6.), with some liking longer texts, and others preferring advice to be presented in different app sections or released only gradually (e.g., as tutorials, as new tips, or feedback on CO readings) (“*it’s a lot of information but it also gives the app a level of seriousness, and when you’re quitting smoking it’s serious”—P1; “this still looks very prototypey, [...] is very texty so like I’m not sure that lots and lots of people will bother reading all of this stuff and it’s also kind of hidden, […] you wouldn’t know that it’s there.”—P2*). Use of images or graphics, including traffic-light imagery for feedback, was preferred to longer texts, but attention was drawn to the need for color-blind-friendly designs. Finally, regarding onboarding and registration (5.7.), some participants were interested in creating a detailed profile at registration, especially if it could help to tailor advice and feedback (“*it could be like you know you create an account for this and all your data gets stored you know, so it’s sort of personalized to you”—P1*). Participants also expected to see some tutorials on CO testing to ensure that they completed the test correctly.

## 4. Discussion

### 4.1. Summary of Findings

This study explored views and preferences of smokers towards a potential new type of cessation aid—a CO Smartphone System (CSS) comprising a personal CO monitoring device and associated smartphone apps. Four sets of observations emerged. First, participants were interested in using CSSs, especially as a novel quitting aid and a tool for self-discovery. They also tended to have high expectations towards CSSs, and articulated a range of desired features and qualities of CSSs. Secondly, satisfaction with the personal CO monitoring device may strongly influence continued engagement with CSSs. Thirdly, while the use of CSSs was expected to motivate cessation, smokers also highlighted potential unintended negative consequences, such as offering reassurance for continued smoking or hindering quitting efforts. Fourthly, notable individual differences emerged in the underlying motivations for, and expected impact and patterns of CSS use, which could have important implications for the design and evaluation of future CSSs.

### 4.2. Implications for Development and Evaluation of CSSs in the Future

Certain functionality and features were commonly mentioned as desired in CSSs. The personal CO monitoring device was expected to be accurate, visually appealing, and convenient to use, which may require the implementation of wireless connections to smartphones in the future. In the meantime, studies or programs using existing devices may need to appropriately manage expectations of potential users at enrolment. Moreover, core features of associated apps should include (1) an easy and quick CO testing journey, (2) clear and visual presentation of CO results, which is in line with prior research [35], (3) and which should be accompanied by relevant and encouraging feedback and advice, as well as (4) a possibility to collect contextual data on CO readings that could help to understand the results and adjust future behavior. Features (3) and (4) could be delivered through Just-in-Time Adaptive Intervention (JITAIs) designs, which aim to offer timely and relevant support that is informed by users’ states and circumstances assessed through active or passive data collection via the app [55]. The effectiveness of these will need to be assessed in further research. Finally, attractive visual designs of apps have been highlighted as desired qualities in prior research [41].

Observations from the current study also suggest that some smokers, including those who are motivated to quit, may interpret feedback from CSSs as demotivating or permissive of smoking, as long as they are not scoring in the top range of CO values. CO testing has been used successfully in traditional face-to-face settings [15,25,26], but more research is needed to understand the impact of using such devices with no involvement of healthcare professionals or cessation specialists, and how to mitigate any negative impact that CSS use could have on cessation efforts.

Furthermore, these findings suggest that certain individual differences may not only impact on smokers’ preferences for CSS features, but also on engagement. A recent review [45] found that socio-demographic and psychological characteristics, including motivation and prior experiences of digital programs, may influence engagement with digital behavior change interventions. The present study echoes these findings, but also suggests that additional, behavior-specific factors may need to be assessed and considered when developing and evaluating CSSs. These should include smoking patterns (especially intensity and regularity of smoking), preferences for quitting approaches and methods (e.g., levels of assistance required or timeframe), the underlying motivation for CSS use (e.g., preferences to record high or low CO readings), and use preferences (timing and location of testing, and device sharing). These factors could have a non-trivial impact on levels and patterns of engagement with the program and CO testing, as well as dropout, and hence, hinder the interpretation of trial data. Such considerations may be especially relevant in studies evaluating CSSs during *ad libitum* use, but could also be relevant in studies with more restrictive designs (e.g., pre-determined scheduled of CO testing). It might thus be important to account for such individual characteristics during study recruitment and subsequent analyses.

### 4.3. Strengths and Limitations

As per best practice and guidelines for person-centered development of complex digital interventions [7,8,46], the study involved a mixed methods approach, involving both in-depth interviews, and think-aloud procedures about a range of prompts, including a modern CO monitoring device, as well as existing and prototype apps. This has enabled a more comprehensive needs assessment among potential end-users of CSSs, as well as exploration of a range of pertinent issues related to the development and evaluation of such programs.

This study had several limitations, however, in light of which the findings should be interpreted. First, the sample was relatively small and self-selected, and over-represented smokers who would be interested to use digital cessation aids, or engage in cessation in the near future. Therefore, the findings may not generalize to the wider population of smokers. The sample was also over-representing smokers from higher socio-economic status (SES), and who had lower dependence levels. On one hand, smokers from higher SES groups are more likely to be ‘early adopters’ of new technologies [56], such as CSSs. On the other, smokers with greater dependence may find CSS even more attractive and relevant, especially for their efforts at harm reduction and cutting down from higher numbers of cigarettes smoked per day. Future research may therefore need to account for needs and preferences of smokers with lower SES and those with higher dependence levels.

Nevertheless, according to guidelines [57] the sample size was adequate given the exploratory aims of the study and data saturation was reached, with no new themes or ideas emerging from the new interviews [49]. Participants also articulated diverse points of view and preferences, and the study identified a range of design suggestions and new potential barriers to the use of CSSs that could inform future development and research. Secondly, as the project matured we adapted the interview schedule and prompts. However, the core parts of the interviews in both phases followed the same structure, allowing for data synthesis. Finally, the study explored views about hypothetical use of CSSs, with participants not able to use the apps or CO monitor in a real-world scenario. Research shows that potential users may not be able to articulate all of their preferences, predict their own behavior, or provide justification for these [41,58]. Therefore, future studies should employ other research methods (e.g., observational studies in the wild) to verify the impact of implementing the preferred functionality on engagement and effectiveness of CSSs [7].

### 4.4. Future Research Directions

Our findings suggest several possible avenues for further research into CSS. First, there may be merit in more systematically exploring and identifying typologies of potential CSS users and their preferences with a view to either tailoring some functionality and content of a generic app to their profile and preferences, or to creating different versions of CSSs that are targeted to sub-samples of smokers with shared characteristics. Second, more research is needed to ascertain the level of tailoring and targeting needed to attain sufficiently high levels of acceptability, satisfaction, engagement with, and impact of CSSs on smoking and quitting behavior. Third, several theories and interventions could be implemented and tested as part of CSS. For example, prior research has shown as acceptable a contingency management intervention involving financial incentives for abstinence verified using remote, video-recorded CO testing [35]. Furthermore, several theories, such as the extended parallel process model [15,59], could inform more impactful ways of conducting CO testing (as risk communication) and providing feedback and advice within CSS. The effectiveness of these will need to be assessed in further quantitative research. It will also be vital to explore the use of CSSs in the real world and as part of efforts to quit or to cut down, but also to assess benefits and risks of using CSSs without the involvement of healthcare professionals.

Finally, given that still only a minority of smokers accesses evidence-based or digital support with quitting, it would be important to assess preferences, interest in, and uptake of CSS among a wider group of smokers, and to identify feasible distribution channel and schemes (e.g., via pharmacies or workplaces). This could involve, for example, developing and administering a survey based on the current qualitative findings among a larger sample of potential CSS users. Nevertheless, creating CSS in a user-centered manner could, at least in theory, increase chances that such programs would be acceptable and relevant to smokers [7,8].

## 5. Conclusions

Some smokers may be interested in using personal CO monitoring devices that connect to smartphones. However, such smokers tend to have very high expectations and hopes for the programs and the impact they could have on their smoking and quitting behavior, especially for increasing and sustaining long-term motivation to remain abstinent. The major challenges in the field will be creating reliable CO monitoring devices with appealing designs, creating apps that meet often divergent needs of different smokers, and devising research paradigms that will enable meaningful evaluation.

## Figures and Tables

**Table 1:** Characteristics of the interviewed participants.

ID	Sex	Age	Post-16 Education	Employment	Cigarettes per Day	Last Quit Attempt	Ever Quit for >1 Week	Ever Tested Carbon Monoxide Levels before	Ever Used Cessation Apps before	Ever Used Evidence-Based Cessation Support
**1**	Female	34	Yes	Employed (non-manual)	10–15	Never	-	-	-	-
**2**	Female	25	Yes	Student	5	Past year	Yes	Yes, once	Yes	Yes
**3**	Female	31	-	Employed (manual)	10–12	Past year	Yes	Yes, once	-	Yes
**4**	Male	24	Yes	Student	3–20	Past year	Yes	Yes, once	-	Yes
**5**	Female	26	Yes	Employed (non-manual)	10	>1 year ago	Yes	-	-	-
**6**	Female	30	Yes	Employed (non-manual)	10	Past year	Yes	-	-	Yes
**7**	Female	28	Yes	Employed (non-manual)	15	>1 year ago	Yes	-	-	-
**8**	Female	40	Yes	Employed (non-manual)	1–2	>1 year ago	Yes	-	-	Yes
**9**	Male	20	Yes	Employed (manual) and Student	6–20	Past year	Yes	-	Yes	-
**10**	Male	28	Yes	Employed (non-manual)	7–10	Past year	Yes	Yes, >once	-	Yes
**11**	Male	51	Yes	Employed (non-manual)	15–20	Past year	-	Yes, once	-	Yes
**12**	Female	26	Yes	Employed (non-manual)	16	Past year	Yes	Yes, >once	-	Yes
**13**	Male	39	Yes	Student	5–8	Past year	Yes	Yes, >once	-	Yes
**14**	Male	46	Yes	Employed (non-manual)	15	>1 year ago	Yes	-	-	Yes
**15**	Male	29	Yes	Employed (manual)	10	Past year	Yes	-	Yes	Yes
**16**	Male	35	-	Employed (non-manual)	15	Past year	Yes	-	-	-

**Table 2:** Themes 1 to 3 and subthemes together with summary of core findings related to general views and expectations for CO Smartphone Systems (CSSs) and their use.

Theme 1	CO Testing—General Views and Motivation to Use
**1.1.**	**General Views on CSSs**
**1.2.**	**Motivation to use—a novel cessation aid** Potentially helpful at increasing motivation to quit and remain abstinent Monitor and inform about health damages from smoking A long-term companion through the smoking and quitting journeys
**1.3.**	**Motivation to use—other reasons** The ‘quantified self’ Opportunity to learn new things about oneself Willingness to contribute to science Tech gadget; something to show off with friends
**1.3.**	**Concerns over CSS** accuracy of CO testing and possibility to manipulate results anxiety and worry over high results annoyance and demotivation due to lack of sufficient progress ‘moderate’ CO levels reassuring and permitting of continued smoking
**Theme 2**	**Practicalities of CSS Use**
**2.1.**	**Commercial use vs. use as part of study** study: acceptance to record personal details, share CO results, use CSS according to schedule outside of the study: expectations to use ad libitum and anonymously
**2.2.**	**Smoking status and CO testing** preferences for testing: when the results is expected to be low vs. high interest to test and record CO levels across a range of situations and smoking levels
**2.3.**	**Location of use** different preferences to use at home, in private vs. in front of friends and family vs. in public
**2.4.**	**Sharing the device** device is private, not to be shared, vs. interested to share with family and friends
**2.5.**	**Timing and duration of use** morning and evening most likely times for testing, especially for home-only testing different preferences for duration of CSS use (only during a quit attempt vs. long term to document smoking and quitting journey)
**2.6.**	**Barriers to CSS use** annoyance or inconvenience of blowing into the device annoyance or inconvenience of needing to connect the device to a phone dislike for carrying around or displaying the cable anticipated embarrassment to test in public limited battery life low relevance for light smokers or abstainers
**Theme 3**	**Factors Potentially Affecting Preferences, Views and Engagement with CSSs**
**3.1.**	**Smoking profile** patterns of smoking (regular vs. irregular) perceived role of smoking (e.g., habit, mood regulation, socializing) dependence levels
**3.2.**	**Barriers to quitting** motivation self-efficacy and capability to remain abstinent, manage cravings other concerns, e.g., weight gain
**3.3.**	**Views on, and plans for quitting** timing of a quit attempt (near vs. distant future) preferred levels of support (e.g., assisted vs. unassisted) approach to quitting (cutting down vs. abrupt cessation)
**3.4.**	**Prior experience with digital programs and user digital behaviors** preferences for features found and enjoyed in other apps extending behaviors with other apps to other apps
**3.5.**	**Prior experience with CO testing**

**Table 3:** Design suggestions for personal CO monitoring devices and associated apps.

Theme 4	Personal CO Monitor: Features and Qualities	
**4.1.–4.2.**	Small size and light weight Wireless connection Rechargeable batteries Possibility to take CO test and temporarily store results without needing to connect to smartphone for each individual test Possibility to display the result on the device Option of different colors Case provided to fit all necessary items (e.g., cables)	
**Theme 5**	**App: Features and Qualities**	
**5.1.**	**CO testing and display of CO results**	
**5.1.1.**	**CO testing journey**	Immediately accessible on app launch Quick and easy testing procedures Clear presentation of a numeric result (in ppm)
**5.1.2.**	**Feedback on CO results**	Presentation of the result on the scale or color-coded Relevant feedback (e.g., health impact) Encouraging advice on lowering the CO levels
**5.1.3.**	**Recording contextual data**	Possibility to collect contextual data on CO readings (e.g., timing and number of cigarettes smoked, levels of urges and stress)
**5.2.**	**Interactive infographics**	Long-term record of CO results Interactive display (zooming in/out, changing time scales) Displaying CO results against targets and thresholds Displaying CO results together with contextual data recorded
**5.3.**	**Factual content**	Information and advice on CO and CO testing, Advice on quitting and cutting down, managing CO levels
**5.4.**	**Additional features**	Customizable reminders to take CO tests Possibility to set targets and goals for CO levels Rewards for reaching targets (in-app or external, e.g., diplomas) Sharing CO results on social media or with selected persons Multimedia demonstrating CO testing procedure
**5.5.**	**External expert support**	Possibility to contact a healthcare professional when concerned Possibility to share CO results with clinicians as part of quitting Integration with traditional cessation interventions
**5.6.**	**General app qualities and Information architecture**	Key and interesting information presented in bite-sized, short communications at different stages of the app Longer text (e.g., advice) available for optional browsing Skippable content and options to re-visit content Use of visuals and imagery to convey information or feedback Imagery and colors friendly for visually-impaired users
**5.7.**	**Onboarding and registration**	Registering with personal details for the study, with option to remain anonymous for commercial use Creating detailed profile supporting personalization Tutorial with key information and advice on CO, CO testing and app use presented at the start, but available on request

## Appendix A

**Table A1:** Interview topics covered in 2016 and 2017 interviews.

	Interview Focus and Specific Topics	Method and Prompts
Needs assessment	Part 1: Participant profile and background information (about 5 min) Brief discussion about past and current smoking and quitting history Prior use of any digital programs (e.g., apps, devices) Any experience with CO monitors and testing in the past	Open-ended questions + verbal prompts *No visual materials or prompts*
	Part 2: Assessment of needs and expectations for CO testing (about 10 min) Interest in CO testing? Why? Interested to use CO Smartphone System? Why? Expectations/general views on CSS Expectations towards CO monitor and associated apps Desired functionality/features of CO monitor and associated apps Expectations for CSS use (When?/Where?/How?/Why?) Readiness to share results (With whom? How?) Reminders (Email, Push notifications, Text) Expectations for information/advice (topic, location, timing of delivery)	Open-ended questions + verbal prompts *No visual materials or prompts*
Demonstration	Part 3: Views and reactions to personal CO monitor General views on iCO^TM^ Smokerlyzer^®^ Designs/looks Size Features and functionality Battery life (about 200 tests or 3 years) Suggestions for improvement	Open-ended questions + verbal prompts *Visual prompts used: iCO^TM^ Smokerlyzer^®^* *No app shown*
User testing of apps or functioning app prototypes	Part 4A (only in 2016): Views on, and reactions to, available apps or app prototypes developed to work with a personal CO monitor (about 15 min). Overall impressions (Likes and Dislikes) Whether would like to use it (when? where? how often?) User journey through the app Journey to complete CO testing Displaying CO results history Other content, information and advice Other issues (language, terminology, amount of texts) Views on ease of use How to improve? Discussion of additional suggestions and possible features (e.g., sharing results, using reminders, setting targets, scheduling testing, etc.)	Usability tests (exploring the apps naturally, navigating the different content and features) + think-aloud procedure *Visual prompts used:* *Smokerlyzer^®^ app by Bedfont^®^* *CO Monitor App prototypes (V1–V2) and designs developed for UCL*
	Part 4B: (only in 2017): Usability testing of CO Monitor app (V3) developed for UCL and discussion of piloting use of iCO^TM^ Smokerlyzer^®^ and CO Monitor App V3 at home *Specific prompts as in 4A above*	Usability testing and think-aloud. *Visual prompts used:* *CO Monitor app (V3) developed for UCL*

**Table A2:** Interview prompts (example screenshots presented) of existing and prototype apps and designs to work with personal CO monitors.

	2016		2017
	**Bedfont^®^ Scientific Ltd. Smokerlyzer^®^ App**	**UCL CO Monitor App (V1)**	**UCL CO Monitor App (V3)**
**Think-aloud of functioning apps or app prototypes**			
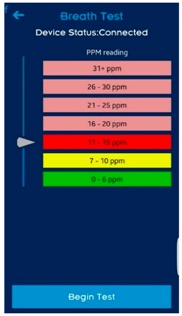
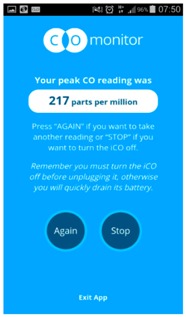
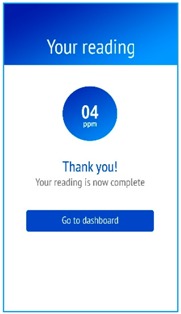
	**UCL CO Monitor Interactive Design (V2)**	**UCL Different CO Monitor Designs**	
**Think-aloud about click-through or static designs of new app prototypes**			N/A
